# Therapeutic application of hydrogels for bone-related diseases

**DOI:** 10.3389/fbioe.2022.998988

**Published:** 2022-09-12

**Authors:** Xiyu Liu, Shuoshuo Sun, Nan Wang, Ran Kang, Lin Xie, Xin Liu

**Affiliations:** ^1^ Third School of Clinical Medicine, Nanjing University of Traditional Chinese Medicine, Nanjing, China; ^2^ Department of Orthopedics, Nanjing Lishui Hospital of Traditional Chinese Medicine, Nanjing, China

**Keywords:** hydrogels, bone-related diseases, tissue engineering, biocompatibility, biodegradability

## Abstract

Bone-related diseases caused by trauma, infection, and aging affect people’s health and quality of life. The prevalence of bone-related diseases has been increasing yearly in recent years. Mild bone diseases can still be treated with conservative drugs and can be cured confidently. However, serious bone injuries caused by large-scale trauma, fractures, bone tumors, and other diseases are challenging to heal on their own. Open surgery must be used for intervention. The treatment method also faces the problems of a long cycle, high cost, and serious side effects. Studies have found that hydrogels have attracted much attention due to their good biocompatibility and biodegradability and show great potential in treating bone-related diseases. This paper mainly introduces the properties and preparation methods of hydrogels, reviews the application of hydrogels in bone-related diseases (including bone defects, bone fracture, cartilage injuries, and osteosarcoma) in recent years. We also put forward suggestions according to the current development status, pointing out a new direction for developing high-performance hydrogels more suitable for bone-related diseases.

## 1 Introduction

The skeletal system is closely related to the movement of the body and its defenses. It may manage basic human physiological functions, which help maintain homeostasis in the body’s internal environment and store minerals (Khiabani et al., 2021). The destruction of the skeletal system leads to endless consequences. Bone-related diseases are mainly associated with a variety of factors, such as trauma, infection, and age (Loeffler et al., 2018). Severe bone damage cannot heal on their own, significantly affecting the life quality of patients (Yue et al., 2020). Traditional surgical treatment methods mainly include prostheses implantation and bone transplantation. Despite their clinical efficacy, they might have side effects such as infection and pain and have disadvantages such as high surgical costs and the need for additional surgery (Kretlow and Mikos, 2007; Khan et al., 2008; Liu et al., 2017; Yue et al., 2020). Of more than two million bone grafts performed worldwide annually, delayed healing occurs in more than 20% of patients. Regrettably, there is currently no satisfactory bone grafting protocol (Chen et al., 2012; Yu et al., 2020; Yue et al., 2020). Up to now, the dominating alternative has been the use of donated, the commonly used allografts have only weak effects of bone induction and bone regeneration. Nevertheless, the development of autologous bone grafting is constrained by factors related to the morbidity of the donor site and the insufficient volume of graft material (Kurien et al., 2013; Gibbs et al., 2016). Therefore, the emergence of new technologies or modifications to existing treatment modalities is urgently sought to provide individuals with utmost care and help patients return to their everyday lives (Nallusamy and Das, 2021).

Tissue engineering (TE) facilitates the creation of three-dimensional (3D) substitutes that closely resemble human tissue to restore and maintain the integrity of tissue structure and physical enginery, which holds promise from repair to human tissue regeneration and individual health restoration (Ghosal and Kaushik, 2020). Combining TE technology with orthopedics is expected to bring unprecedented benefits. Bone TE (BTE)-related techniques are often used in some cases of bone-related diseases, such as blocked bone regeneration (Li J. J. et al., 2018). Cells, growth factors, scaffolds and bioreactors are main components of BTE (Vermonden et al., 2012; Li J. J. et al., 2018). In BTE, scaffolds are key elements for building biological structures that resemble natural bone. Recently, a satisfactory 3D scaffold design with numerous advantageous features has been proposed (Nallusamy and Das, 2021).

To date, polymeric materials widely used in the biomedical field have attracted much attention due to their characteristic superiorities such as biocompatibility, processability and low cost (Henry et al., 1998; Sigfridsson et al., 2009; Tuan-Mahmood et al., 2013). Nowadays, polymeric patches of ingeniously designed with stimuli-responsive properties are widely used as biomedical scaffolds in biomedical field. Currently, materials associated with such polymers mainly include hydrogels, microneedles (MNs), microcapsules, microspheres, and fibers, which exhibit excellent biocompatibility and biodegradability (Shahiwala, 2011).

In BTE, hydrogels are prominent among the numerous available biomaterials that constitute scaffolds (Nallusamy and Das, 2021). A unique advantage of the hydrogels must be pointed out, their specific porous structures similar to the extracellular matrix (ECM) can serve as carriers for various growth factors (Ulery et al., 2011; Buwalda et al., 2016; Liu et al., 2018). Moreover, their soft texture reduces surrounding inflammatory responses (Buwalda et al., 2016). In consequence, hydrogels are quite appropriate as scaffold-related materials for BTE (Yue et al., 2020). This article reviews the application of hydrogels in bone-related diseases in recent years, involving bone defects, fractures, cartilage damage and osteosarcoma. The above research hopes that this contribution will further guide scientists to develop high-quality hydrogels suitable for bone regeneration.

## 2 Hydrogels

Hydrogels are highly hydrated 3D networks of hydrophilic polymers. The cross-linking of polymer chains enables them to absorb and retain large amounts of water in a porous structure (Gan et al., 2001; Loh et al., 2011; Nguyen et al., 2011; Barouti et al., 2016, 2016; Gibbs et al., 2016; Jiang et al., 2019; Yue et al., 2020; Lin et al., 2021; Oliveira et al., 2021; Liu et al., 2022). Due to the similarity between hydrogels and tissue ECM, they are widely used in the biomedical field as a biocompatible polymeric material (Caló and Khutoryanskiy, 2015). In accordance with many biological materials, hydrogels can be divided into two categories: natural polymers and synthetic polymers (Ahmed, 2015; Zhu et al., 2022). So far, many different kinds of hydrogels have been applied in the pharmaceutical industry. Hyaluronic acid, gelatin, alginate, dextran, chitosan, collagen, elastin and albumin are all natural polymers that can be complexed into hydrogels (Rivory et al., 1994; Bouhadir et al., 2001; Liu et al., 2006; McDaniel et al., 2010; Yue et al., 2020). These materials generally have good biocompatibility and biodegradability, combined with practicality and durability. Hydrogels produced through their self-assembly or cross-linking have been widely exploited in drug delivery systems (He et al., 2018; Zhu et al., 2022). In addition, smart delivery nanoplatforms constructed with some natural polymers have been used as hydrogel drug depots (Zhu et al., 2022). However, these natural polymers have inconsistent hydration and elastic properties. Common biosynthetic materials include poly (ethylene glycol) (PEG), poly (N-isopropylacrylamide) (PNIPAAm), polycaprolactone (PCL), poly (L-glutamic acid) (PGA), polypropylene fiber (PPF), and polyvinyl alcohol (PVA) (Yue et al., 2020). Synthetic polymers-derived hydrogels offer controllability and reproducibility to improve consistency and alter properties (Ahmed, 2015; Gibbs et al., 2016). Whereas, synthetic biomaterials’ biocompatibility and safety are weak, that own lower biological activity than natural biomaterials (Amini and Nair, 2012). Both natural and synthetic polymers have pros and cons. In order to take full advantage of their excellent properties, several materials can be used in combination (BaoLin and Ma, 2014; Oliveira et al., 2021). In BTE, the application of hydrogels has advantages and limitations; thus, it is often applied to specific scenarios according to the characteristics of each hydrogel. It is particularly emphasized that composite hydrogels are often prepared to reduce the limitations and maximize the advantages. In each bone-related disease, we will exemplify the specific applications of representative hydrogels in detail.

### 2.1 The evolution of hydrogels

Its evolution has gone through several generations (Nallusamy and Das, 2021). In 1960, the porous polymers named poly (2-hydroxyethyl methacrylate [HEMA]) were used to fabricate contact lenses (Wichterle and Lím, 1960; Nallusamy and Das, 2021). The first-generation hydrogels are mainly about a single chemical polymer network. Second-generation hydrogels refer to stimulus-responsive hydrogels that respond when the environment changes (Hodgson et al., 2017; Nallusamy and Das, 2021). The third-generation hydrogels established cross-linking methods with physical interactions, which were expected to tune the properties (Buwalda et al., 2016; Nallusamy and Das, 2021). Up to now, the fourth-generation hydrogels have been developed into smart hydrogels, which possess strong stability and stable performance to achieve more accurate targeted delivery (Buwalda et al., 2014; Nallusamy and Das, 2021).

### 2.2 Main properties of hydrogels

Hydrogels have been widely used in tissue engineering due to their unique performance advantages, such as softness, water absorption, tunable mechanical strength, and controllable degradability. Especially in BTE, hydrogels have promise as functional scaffolds for growth factor transport and cell adhesion (Yue et al., 2020).

**FIGURE 1:**
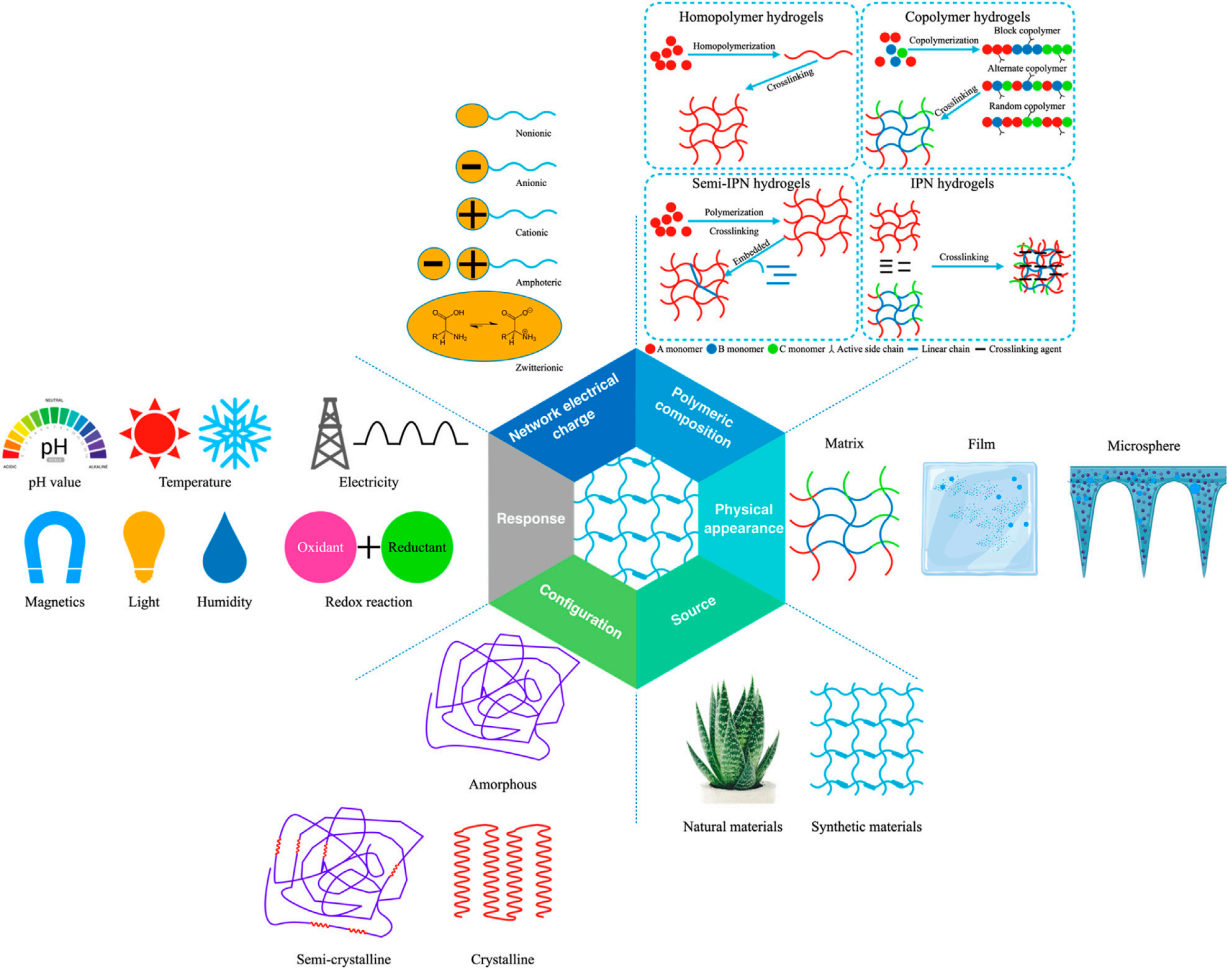
Classification of hydrogels based on properties.

Hydrogels have a great characteristic of absorbing a large amount of water or biological liquid, among many material forms. This characteristic gives hydrogels excellent softness, biocompatibility, and hydrophilicity and shows a good fit with the softness of living tissue. This beneficial characteristic is also a prerequisite for biological application (Caló and Khutoryanskiy, 2015; Kondiah et al., 2016; Nakka and Mungray, 2016; Chai et al., 2017; Nallusamy and Das, 2021). In addition, the structure and coordination of hydrogels in their hydrated form guarantee structural strength and also facilitate the flow of liquids, metabolites, nutrients or agents (Khiabani et al., 2021). The unique physical properties of hydrogels make them the workhorse of drug delivery systems (Chai et al., 2017). The porosity of the hydrogel is used to encapsulate the drug into the gel, while the diffusion coefficient of the molecules in the hydrogel network determines the drug release rate (Li and Mooney, 2016). Notably, the properties of hydrogels can be adapted to slow drug release patterns, maintaining higher drug concentrations where they are administered for prolonged periods (Hoare and Kohane, 2008; Narayanaswamy and Torchilin, 2019). It can also be matched with environmental conditions to release and degrade controlled drugs (Hunt et al., 2014; Li and Su, 2018; Mantha et al., 2019; Oliveira et al., 2021). It is worth mentioning that, as “smart gels”, hydrogels are exceptionally sensitive to external stimuli such as ionic strength, pH and temperature, demonstrating their high efficiency (Vashist et al., 2014). More importantly, the hydrogels are durable and show high stability during storage. Based on their different properties, hydrogels have been classified into the following categories (Figure 1) (Lee and Shin, 2007; Ahmed, 2015; Thakur and Thakur, 2018; Ghosal and Kaushik, 2020; Yue et al., 2020; Nallusamy and Das, 2021). It should be pointed out that, according to different classification methods, we mainly classify from source, physical appearance, polymeric composition, network electrical charge, response, and configuration.

### 2.3 Main influencing factors for the preparation of hydrogels suitable for bone TE

The 3D space formed by the scaffold material in BTE provides a region for cells to survive. Integrins are heterodimeric receptors in cell membranes that connect cells to substrates by binding to adhesion proteins on the biomaterial surface (Clark et al., 2020). Among them, the key factors regulating cell behavior in biomaterials are listed as follows: chemical component, mechanical property, morphology and hydrophilia. According to information, ideally beneficial scaffold material should be matched to specific conditions, as listed below: 1) excellent biocompatibility and degradability; 2) good porosity and 3D structure (Yue et al., 2020). For example, many macromolecules structurally similar to the ECM are ordinarily used to prepare hydrogels to modulate the properties of attached cells. Such molecular materials mainly contain collagen, fibronectin and laminin (Yue et al., 2020). Therefore, the preparation methods of hydrogels should be integrated with superior materials and sophisticated technologies to make them more suitable for treating bone-related diseases.

In BTE, various mechanical properties (tension, compression, bending, swelling, deswelling, indentation, pore diameter, pore shape, and interpore connectivity) should be considered to fabricate suitable scaffolds (Pacifici et al., 2018). The mechanical strength of most bioactive hydrogels is not strong, which limits the application of hydrogels in BTE (Utech and Boccaccini, 2016; Xue et al., 2021). Based on these, It is crucial to prepare hard and tough hydrogels with tunable mechanics. Processing should be carried out to improve the properties according to its imitations. Advanced techniques in different fields can be used to process hydrogels into desired structures. For example, 3D bioprinting is highly representative (Ansari et al., 2017; Pacifici et al., 2018). We can incorporate inorganic fillers into the polymer matrixes or perform chemical modification techniques during processing to improve mechanical stability. The properties of tensile strength, shape recovery, and energy dissipation can be improved by adding extra physical cross-linking points (Utech and Boccaccini, 2016). Hydrogel matrices can enhance strength and stiffness by incorporating nanotechnology. Additional ions can also be appended to overcome the drawbacks (Kilpadi et al., 2001; Hoppe et al., 2011). Although the addition of inorganic materials can effectively improve the physical structure of hydrogels, there are also some shortcomings, such as the dissolution of harmful substances into adjacent cells and the mechanical stability needs to be further improved. Rauner et al. (2017) achieved significant progress in the development of mechanically tunable hybrid hydrogels by exploiting the changes under enzymatic reaction to obtain hydrogels with extremely strong hardness and tenacity. So far, there is a view that it is crucial to focus on the inherent structure of bone, which helps to optimize the mechanical properties and provides a new perspective for designing bionic materials that aid in bone regeneration (Xue et al., 2021).

Nanoparticles, ranging in diameter from 1 to 100 nm, are solid colloidal particles composed of natural synthetic or semi-synthetic polymers. They are commonly used as reservoirs for nanoparticle systems and as carriers for drug delivery systems (Gan et al., 2019). In exploring emerging drug replacement therapy methods, nanoparticle drug delivery systems have two outstanding advantages, which can not only enhance drug penetration, but also prolong the effective circulation time of drugs in the body (Wu et al., 2022). It is worth mentioning that some macromolecular nanomaterials composed of nanoparticles (such as nanosheets, nanotubes, mesoporous materials, etc.) are often used as nanoplatforms to carry drugs to treat diseases (Yan J. et al., 2021; Wang Y.-C. et al., 2021; Zhu et al., 2021). To date, nanomaterials have been widely used in various fields of medical treatment (Zhu et al., 2018, 2019). With its superior physical and chemical properties, it has been extensively explored and developed in various therapeutic modalities (Zhu et al., 2022). Based on the different configurations of hydrogels that have been used in BTE, the aforementioned fusion of nanotechnologies will help provide state-of-the-art strategies for facing bone-related diseases with hydrogels. The incorporation of nanoparticles into the polymeric matrix could lead to improving the mechanical and electrical features for obtaining satisfactory therapeutic efficacy (Zhu et al., 2022).

The pore size of the hydrogels also plays a key role in guiding the cells into the hydrogel networks. It is necessary to control the appropriate pore size to prepare hydrogels with high performance related to bone repair. At present, there are mainly three types of apertures. Different pore sizes are associated with the appearance of different cellular behaviors. Hydrogels with small pore sizes (2–50 nm) utilize large enough surface area to load drugs, resulting in a good therapeutic effect (Huang et al., 2015). Hydrogels with a moderate pore size (10 μm) can promote the formation of hydroxyapatite and introduce bone morphogenetic proteins by increasing the exchange rate of calcium, magnesium, zinc and other mineral ions (Schröter et al., 2020). Large pore size (above 100 μm) can significantly accelerate cell migration and adhesiveness (Li Y. et al., 2017). Although hydrogels with diverse pore sizes have multiple functional benefits. Nevertheless, they still exist morphological limitations. To address these types of problems, 3D printing and other techniques related to elaborate processing have been exploited far and wide to precisely fabricate hydrogels with matched mechanical properties, tailored porosity and structure for BTE (Gauvin et al., 2012; Chen et al., 2020; Matai et al., 2020; Wang et al., 2020, Wang et al., 2021 C.).

### 2.4 Preparation methods of hydrogel

In general, hydrogels are mainly associated with two types of cross-linking modes: physical cross-linking and chemical cross-linking (Yue et al., 2020). Physical hydrogels’ attachment modalities include ion interactions, electrostatic interactions, hydrogen bonds, hydrophobic interactions and crystallization (Nabavi et al., 2020; Neves et al., 2020; Khiabani et al., 2021). Methods for synthesizing chemically cross-linked hydrogels include Michael addition reactions, Schiff base reactions, Diels–Alder cycloadditions, radical polymerization, and other click chemistry (Neves et al., 2020). In recent years, physically and chemically related triggering conditions have been commonly utilized to fabricate hydrogels (Hennink and van Nostrum, 2002; Hu W. et al., 2019). In physically triggered conditions, light or temperature stimulates hydrogels’ cross-linking. Whereas under chemically triggered conditions, molecular or ionic cross-linking agents can make covalent bonds or coordinative bonds between polymer chains to form steady hydrogel networks (Voorhaar and Hoogenboom, 2016). Among them, hydrogels manufactured under physical trigger conditions have some outstanding features of clinical application. For example, their mild formation temperature and low toxicity in cross-linking reaction are extremely reliable and trustworthy. The hydrogels prepared by this method have great potential application value in pinhole bone defects and fractures. Whereas chemically triggered hydrogels are covalently cross-linked *via* monovalent, bivalent, or multivalent, they tend to intervene in those hard and large bone defects (Xue et al., 2021).

#### 2.4.1 Physically cross-linked hydrogels

The gelation process of physically cross-linked hydrogels occurs under mild conditions, the interactions involved are mainly ionic. The triggering conditions are often related to temperature (Nonoyama et al., 2020; Xue et al., 2021). Interestingly, ions also play critical roles in maintaining body homeostasis (Zhang et al., 2021b; 2021c). For example, natural polysaccharide hydrogels are usually prepared adopting the principle of ionic interactions. Such hydrogels have valuable potential applications in BTE, effectively supporting the adhesion, proliferation and differentiation of angiogenic and osteoblasts cells (Han et al., 2013). Many injectable hydrogels are also formed through physical interactions. For example, Hou et al. (2015) researched a self-assembled injectable hydrogel linked by hydrogen bonding with striking biocompatibility, biodegradability, and sustainable release of biomolecules, which could interact with different types of biomolecules for BTE. Regarding temperature-triggered hydrogels, thermosensitive hydrogels are fabricated by covalent cross-linking thermo-responsive chains into polymers. PNIPAAm, poly (N,N-diethyl acrylamide), poly (lactic-co-glycolic acid)-PEG and soluplus are some of the commonly-used thermo-responsive polymers (Li X. et al., 2017; Wu et al., 2017; Ekerdt et al., 2018; Hanyková et al., 2020; Xue et al., 2021). Temperature-responsive polymers have great potential for application in drug delivery, of which PNIPAAm is a typical representative due to its ability to reversibly swell in aqueous solutions (Vadnere et al., 1984; Mortensen and Pedersen, 1993; Khiabani et al., 2021). However, pristine hydrogels’ adjustable mechanical capabilities and rheological properties are limited. Hence, bonding PNIPAAm with other polymer chains can enhance its stabilities and mechanical properties (Li J. et al., 2018). The survey report showed that hyaluronic acid (HA), elastin-like protein (ELP), alginate (AAlg), gelatin, monoacryloyloxyethyl phosphate (MAEP), furfurylamine grafted chondroitin sulfate (ChS-F) and hydroxyapatite (HAp) incorporated into PNIPAAm could effectively improve mechanical strength, thermo sensitivity, degree of crystallization, swelling ratio and other related properties (Khiabani et al., 2021).

#### 2.4.2 Chemical cross-linked hydrogels

Chemical reactions can significantly improve the control of flexibility and precision associated with crosslinking, stabilize the hydrogel matrix, enhance mechanical properties and increase stability (Zhang and Khademhosseini, 2017; Xue et al., 2021). Moreover, for the application of hydrogels in substance delivery, the chemically cross-linked form may supply more efficient release dynamics (Khiabani et al., 2021). To date, numerous chemical cross-linking modes have been exploited in hydrogel systems, which comprise small molecule cross-linking, photo-induced cross-linking, and enzyme-induced cross-linking (Xue et al., 2021).

Small-molecule crosslinkers include tannic acid (TA), glutaraldehyde, dopamine, genipin. Since glutaraldehyde has limited use due to toxic side effects on cells and tissues, TA, dopamine, genipin, and caffeic acid are often used as ideal substitutes in polymer networks to enhance the biological properties of materials (Oryan et al., 2018; Xue et al., 2021). Advanced hydrogels cross-linked by different molecular reagents show many compelling merits, including extended selectivity, rapid gel-formation ability, and tunable mechanical properties (Xue et al., 2021).

Photo-triggered hydrogels are triggered by specific wavelengths of light (ultraviolet (UV) or visible light) to induce the formation and morphological changes of 3D networks of gel (Zhu H. et al., 2020). Multiple nature/synthetic-derived polymers are modified with light-responsive cross-linking groups. For example, natural sources such as collagen, HA, and gelatin polymers are modified to obtain such hydrogels. The photo-crosslinking method facilitates the formation of *in situ* gels and achieves precise spatio-temporal control. Relevant optical properties include wavelength, distance, power, and exposure time of the applied light (Zhang K. et al., 2021; Kirillova et al., 2021). It is worth noting that the photo-crosslinking process of the gel must exist photoinitiators, which determine the wavelength of exposure and the quality of gel formation (Xue et al., 2021). However, toxic photoinitiators affect the cell-loaden type and biocompatibility and destroy the hydrogel’s friendly internal environment, limiting the hydrogel’s application in BTE (Balakrishnan and Banerjee, 2011).

Due to the potential toxicity of small-molecule chemical crosslinkers and residual reagents, other methods should be sought to avoid this tricky problem. Enzymatically cross-linked hydrogels have excellent biocompatibility, tunable stiffness and rapid gelation (Akhtar et al., 2016). Thus, focusing on enzymatically catalyzed gel-formation may point a hopeful direction for the subsequent development in BTE. Among them, a representative example is a cross-linking reaction initiated by horseradish peroxidase (HRP) (Xue et al., 2021).

## 3 Hydrogels in bone diseases

Combined with tremendous improvements in the preparation of hydrogels, which exhibit excellent physical and chemical properties, making them cutting-edge biomaterials. How these materials can ultimately be applied clinically and tuned for specific clinical applications has been investigated (Zhang and Khademhosseini, 2017). During these decades of progress, an increasing number of novel hydrogels have been applied to targetable drug delivery and treatment of diseases in BTE (Yu et al., 2020; Yue et al., 2020).

As the scaffold material in BTE, hydrogels mainly play the following functions: 1) to transport the cells to specific sites; 2) to facilitate interactions between cells and biomaterials, and promote cell attachment; 3) to guarantee cell survival, vascularization, proliferation, and differentiation (carriers for protection and delivery of substances); 4) to control tissue structure and function (essential subunits providing mechanical strength); and 5) to ensure safety (negligible inflammation or toxicity) (Langer and Tirrell, 2004; Preethi Soundarya et al., 2018).

In BTE, injectable hydrogels are usually employed, which can be minimally invasive to reach the defect site to fill internal large or irregular defects. Injectable forms of hydrogels can treat deformities of any shape, as well as deliver drugs and immobilize injured bone tissue. Factors such as curvature, pore geometry, pore size, and porosity associated with composite scaffold materials play a key role in bone formation (Zadpoor, 2015). Typically, composite materials are incorporated into hydrogels with the main purpose of maintaining the cohesiveness of the particles both during injection and after delivery to the defect site. In BTE, common examples include Oligo [poly (ethylene glycol) fumarate] and calcium phosphate (apatite), gelatin methacrylate and HAP, cyclic acetal hydrogels and nano-HAP (hydroxyapatite), poly (ethylene glycol) diacrylate and clay, alginate and 45S5 bioactive glass (BG), elastin-like polypeptide collagen and 45S5 BG, et al.(Chang et al., 2010; Patel et al., 2010; Bongio et al., 2011; Wheeler et al., 2013; Zeng et al., 2014; Sadat-Shojai et al., 2015; Utech and Boccaccini, 2016; Nallusamy and Das, 2021). However, prolonged usage of injectable hydrogels can also lead to damage to surrounding tissues, limiting the maintenance of mechanical and biological properties. Fortunately, shape memory and self-healing hydrogels can help solve this problem by preserving the functional level of cells and tissues. Shape memory hydrogels exhibit extremely high tensile strength. Self-healing hydrogels possess both high tensile strength and superior elongation at break (Mehrotra et al., 2020).

**FIGURE 2:**
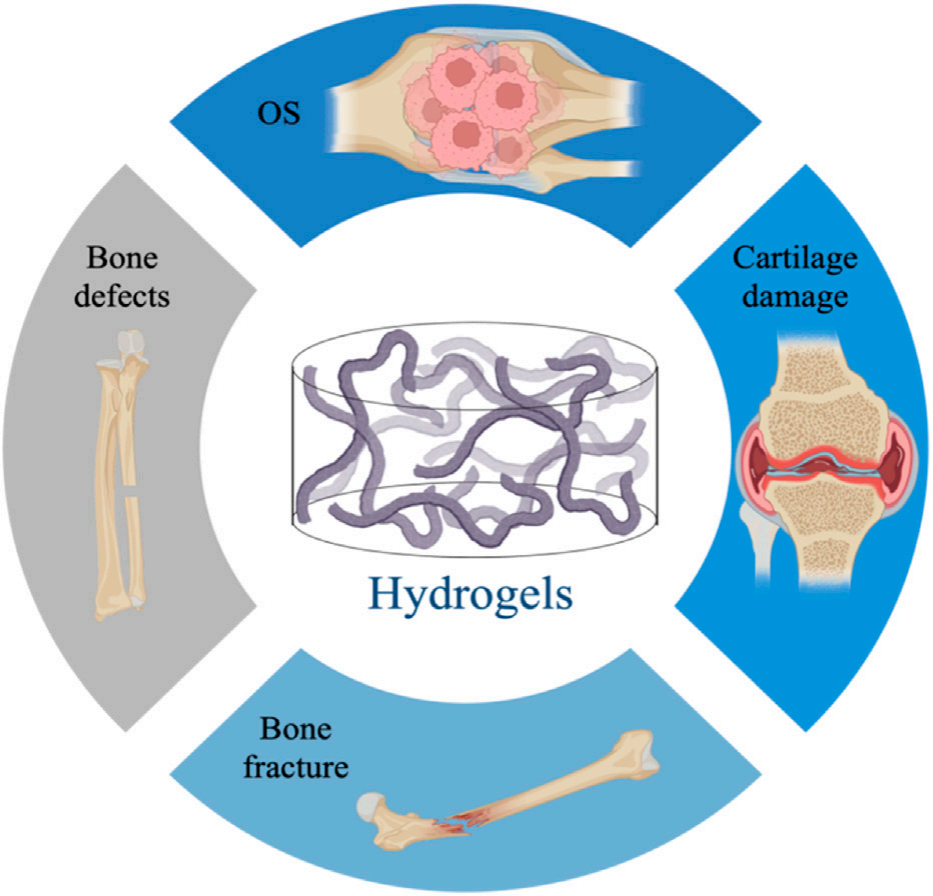
Application of hydrogels in bone diseases, including bone defects, fractures, cartilage damage, osteoarthritis.

The preparation of biomimetic hydrogels in the field of BTE provides a broad prospect for the treatment of bone-related diseases (Xue et al., 2021). Below we describe the application of hydrogels in bone defects, fractures, cartilage injuries, and osteoarthritis, where regeneration of bone loss is a key consideration for their therapeutic goals (Benjamin, 2010) (Figure 2).

### 3.1 Bone defects

Bone defects are mainly severe painful injuries caused by fractures, infections, trauma, tumor resection or skeletal abnormalities (Yu et al., 2020). At present, although autologous bone grafting is still the momentous means of treatment, the availability of the source and the secondary injury it causes limit its widespread use. Bone graft scaffolds are gradually being widely used because they can overcome the above problems and have remarkable clinical application effects (Bauer and Muschler, 2000; Henkel et al., 2013; Perez et al., 2018). Notably, in bone defect repair, the ECM plays an important role as bridging in signal transduction and interchange of material between the regenerating tissue and the original structure. The network structure of the hydrogel can effectively mimic the ECM (Cui et al., 2019; Shang et al., 2021).

**FIGURE 3:**
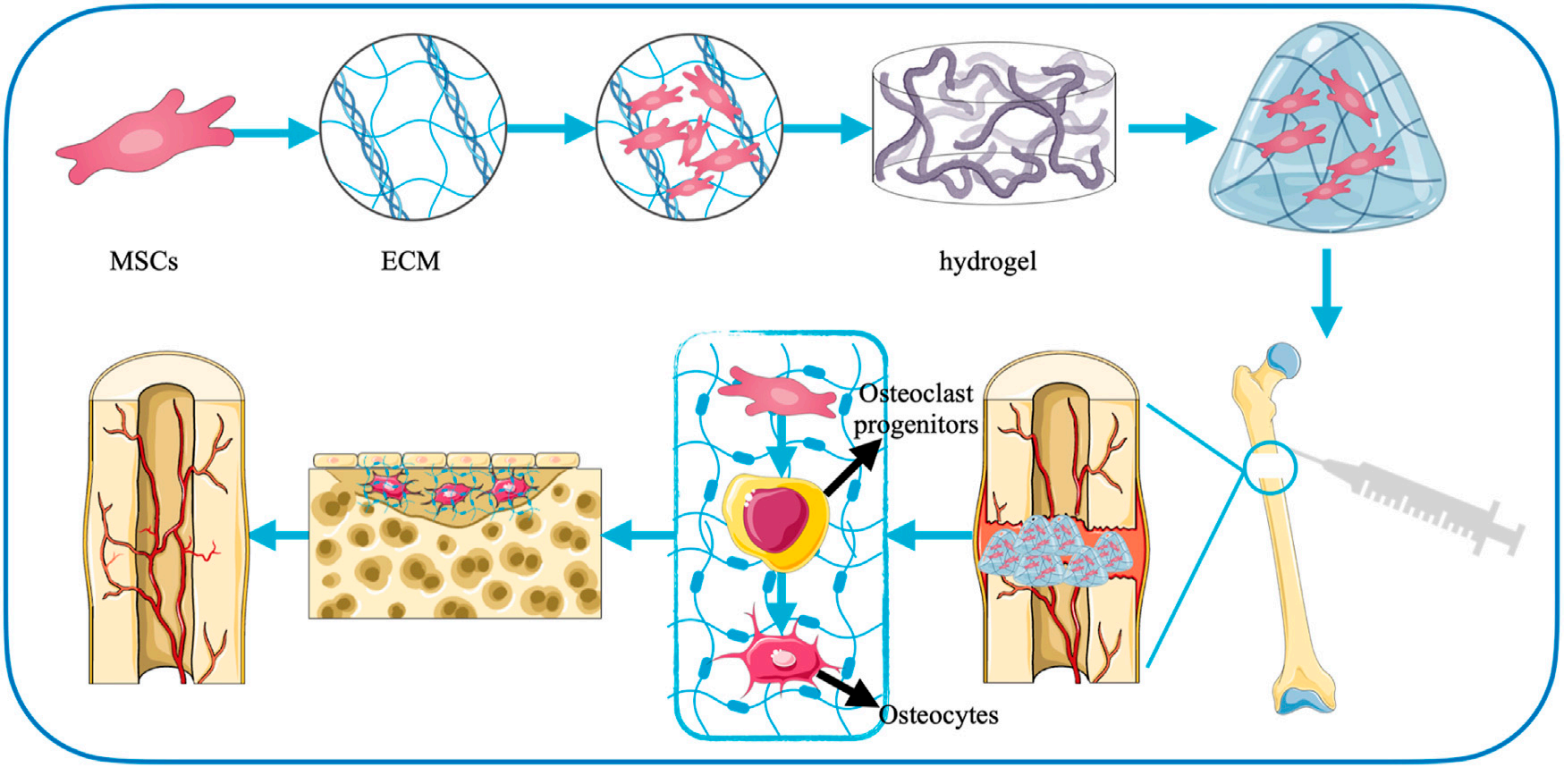
The role of hydrogels in the treatment of bone defects.

Therefore, hydrogel materials have natural advantages as scaffolds for new bone tissue growth, which exhibit great potential in treating delayed bone repairing or wound healing (Figure 3). In addition, the repair of bone defects is actually an osteogenic effect, which is mainly based on the growth, multiplication, and maturation of osteoblasts. When the self-recovery ability is insufficient, growth factors and cells pass through the hydrogels to promote the proliferation and differentiation of mesenchymal stem cells (MSCs) and accelerate bone synthesis (Chaudhuri et al., 2016; Yue et al., 2020). At present, there are various cross-linking influencing substances used to prepare hydrogels suitable for bone defects, mainly mineral ions, thermosensitive polymers, small molecule cross-linking agents, photoresponsive polymers, enzymes, exosomes, and molecular therapies.

Mineral ions are an integral part of maintaining the physiological balance and stabilization of body and can form functional hydrogels by linking with polymer chains to accelerate bone regeneration (Xue et al., 2021). Thus, ionic-based hydrogels are frequently used in bone-related diseases to promote osteoanagenesis. Irons are often incorporated into biomimetic materials to improve blood vessel formation during bone regeneration. Copper can also effectively maintain the bone quantity and accelerate wound repair. The data have shown that adding copper ions to the hydrogels could maintain its network structure for a long time and ensure the sustained-release behavior of biologically active substance (Pontremoli et al., 2018; Xue et al., 2021). Not only that, recent studies have shown that Mg^2+^ has unlimited potential. Its role in promoting bone differentiation and bone regeneration in bone defects is very obvious (Lin et al., 2018; Obata et al., 2019). Pan et al. (2020) incorporated MgO nanoparticles into hydrogels and found that the addition of Mg^2+^ enhanced the osteoblast differentiation of bone mesenchymal stem cells (BMSCs) and accelerated bone tissue regeneration. In addition, a 3D hydrogel scaffold combined with hydroxyapatite/MgO nanocrystal designed by a research group also enhanced the repair of bone defect in diabetic rats.

Thermosensitive polymers (such as PNIPAAm, Soluplus) can be incorporated into the backbone to fabricate highly biocompatible thermoresponsive hydrogels for bone defects requiring minimally invasive surgery (Xue et al., 2021). However, such hydrogels usually have poor mechanical properties and need to be composited with other materials to enhance the strength of the matrix (Ahmed, 2015; Bahram et al., 2016; Fuchs et al., 2020; Ji and Kim, 2021). It has been found that incorporating of HA into thermally responsive hydrogel systems can enhance the physical mechanics performance of the hydrogels. HA becomes the main hydrophilic chain of such hydrogels due to the presence of hydroxyl and carboxyl groups (Ekerdt et al., 2018). Furthermore, HA is also a major part of ECM, which facilitates a range of cellular behaviors *in vivo*. Studies have shown that HA-PNIPAAm polymers are mainly generated by modifying the thiol-terminated PNIPAAm with the pendant vinyl groups of HA-VS (Ekerdt et al., 2018; Xue et al., 2021). And the constructed hydrogels have regulated mechanical behavior adapted to specific stem cell differentiation, for example, which greatly promoted the osteogenic differentiation of BMSCs (Rape et al., 2015).

Small-molecule crosslinkers, including dopamine, nanoclays, genipin and TA, are frequently introduced into polymer networks as ideal substitutes for enhancing the properties of biomaterials. Li et al. (2016) found that introducing metal ions (Fe^3+^) and phenolic hydroxyl groups into dopamine-modified polymers can form dynamic covalent bonds, ultimately enhancing the controllability of hydrogels’ mechanical properties. It must be mentioned that, due to the reversibility of the structure of metal coordination bonds, this hydrogel also has a self-healing capacity. That is to say, this type of hydrogel has both tunable mechanical properties and self-healing ability. As a broad-spectrum hydrogel model, it is not only suitable for soft tissue defects (skin wounds) but also has a remarkable curative effect on bone defects. In addition, the fusion of nanotechnology and hydrogels provides a novel idea for bone defect repair. Introducing nanoclay as the crosslinking agent at the nanoscale into the caffeic acid-modified chitosan system can form versatile, self-rehabilitation hydrogels (Xue et al., 2021). The nanoclay-crosslinked hydrogel shows good osteoinductivity by modulating the Wnt/β-catenin pathway and can be used in arbitrary bone defects. Genipin also acts as a cross-linking agent to enhance the mechanical properties of 3D scaffold. Another study have shown that TA-related hybrid hydrogels could guide bone regeneration in an experimental mouse model (Bai et al., 2020).

Photoresponsive polymer is a kind of polymer that can produce reversible changes in various physical properties under the action of light. Gelatin is a photoresponsive polymer that can be modified by photo-crosslinked groups, mainly containing acrylamide, methacryloyl, and norbornene. Gelatin methacrylate (GelMA) is the most typical photo-crosslinked polymer, which exhibits biological and mechanical properties vary with the degree of methacrylation and hydrogels’ concentration (Zhao et al., 2016). It should be pointed out that GelMA itself has low osteogenic activity; some bioactive components need to be introduced to enhance the bone regeneration ability of GelMA (Xue et al., 2021). Osteogenic growth peptide (OGP) is an active compound that enhances bone repair *in vivo* (Gabarin et al., 2001). Studies suggest that OGP-crosslinked GelMA hydrogels can improve cellular adherence and growth and accelerate the expression of osteogenesis-related genes, promoting the regeneration capacity of new tissue in bone defects. Qiao et al. (2020) prepared a novel hydrogel by utilizing the form of covalent linkage between GelMA and OGP under UV light to enhance the osteogenesis potential of GelMA-based hydrogels. In a rat distal femoral defect model, this hydrogel demonstrated enhanced repair capacity *in vivo* and an accelerated rate of new bone regeneration.

Studies have shown that hydrogels under the action of enzymes can also promote bone repair, showing great application prospects. For example, Xue et al. (2021) prepared an enzymatic hydrogel with chondroitin sulfate (CS) and HA in the presence of H_2_O_2_ and HRP in a specific environment. The researchers conducted *in vitro* and *in vivo* experiments. The results showed that the microenvironment provided by the newly prepared hydrogel is favorable for the osteoblastic differentiation of BMSCs and the bone tissue repairing of rat femur.

In addition to using traditional and common cross-linked polymers to prepare hydrogels with excellent properties for bone defects, many emerging strategies for hydrogel innovation have also been proposed to enrich the field of bone defect treatment. The secretory process of paracrine signaling is closely linked to tissue damage repair (Al-Samadi et al., 2017). Exosomes are essential for the inflammatory response after injury and can promote the regeneration and reconstruction of damaged tissues (Jing et al., 2018). Currently, many studies have focused on loading exosomes in hydrogel systems. It has been indicated that exosomes from human bone marrow-derived MSCs could effectively enhance osteogenic differentiation (Pethő et al., 2018). Animal model studies have also shown that exosomes loaded in hydrogels can help boost osteogenesis and healing of damaged bones in the rat models (Yang et al., 2020). Various studies indicate that this hydrogel exhibits excellent bone repair ability while possessing superior properties (such as strong self-repair ability, high biocompatibility, and low toxicity.) (Pishavar et al., 2021).

Currently, diagnosis and treatment of diseases at the molecular and cellular levels have been explored from multiple perspectives, such as RNA interference (RNAi) technology. It is mediated by microRNAs (miRNAs) and small interfering RNAs (siRNAs) and is an effective strategy for post-transcriptional gene regulation. Nevertheless, the adhibition of RNAi therapies related to bone regeneration has not yet entered the clinical trial stage, so there is still a lot of research space. One of the major difficulties is the inability to achieve sustained release of RNA molecules in the target site of bone defect and surrounding cells. We still lack a suitable carriers to accompany the safe operation and get effective results. A likely approach is to encapsulate RNAi molecules into hydrogels, which can also be delivered in combination with nanoparticle technology (Yu et al., 2020). Nanomaterials, which have been mentioned earlier, are often incorporated into polymer matrices as promising reinforcement materials for next-generation BTE applications (Zhu et al., 2022).

### 3.2 Bone fracture

The bone fracture usually occurs under high-force shock or pressure. Bone formation and growth are crucial for treating bone fractures (such as avulsion, comminuted, and crush fractures) (AI-Aql et al., 2008; Yun et al., 2021). At present, most fractured bone tissue heals itself, while complex fractures require interventions to promote bone repair (Agarwal and García, 2015). Minor fractures can be recovered without surgical intervention, but the recovery period is long and affects the patient’s quality of life (AI-Aql et al., 2008). In contrast, multiple complex fractures have poor recovery and often require invasive surgery (AI-Aql et al., 2008; López et al., 2014). The application of 3D polymer matrix to repair damaged bone tissue in fracture patients is a hot research direction currently (Han et al., 2021; Khiabani et al., 2021).

Using autologous bone and prosthetic implants to improve bone reconstruction can accelerate bone healing and maintain the degree of recovery to minimize surgical intervention. In the process of exploring treatment methods for bone fractures, a research group proposed to use hydrogel-type bone-derived decellularized extracellular matrix (bdECM) and β-tricalcium phosphate (β-TCP) to immobilize 3D-printed polycaprolactone scaffolds. Using three treatments of two different materials (bone-derived ECM, beta-TCP, and a combination of both), the researchers evaluated their performance as materials for inducing fusion in native bone grafts. A porous-structured polycaprolactone (PCL) scaffold was placed in the centre of the rat calvarial defects model. Then, each material was used to fill the gap between the PCL scaffold and the defective bone. The bone formation capacity in the organism was finally assessed by histological analysis. *In vitro* experiments, the properties of the materials were also evaluated with MG63 cells. The results showed that the bone-derived ECM-β-TCP mixture showed faster bone formation in rats and was an ideal osteogenic promoter for the therapy of bone fractures (Yun et al., 2021).

**FIGURE 4:**
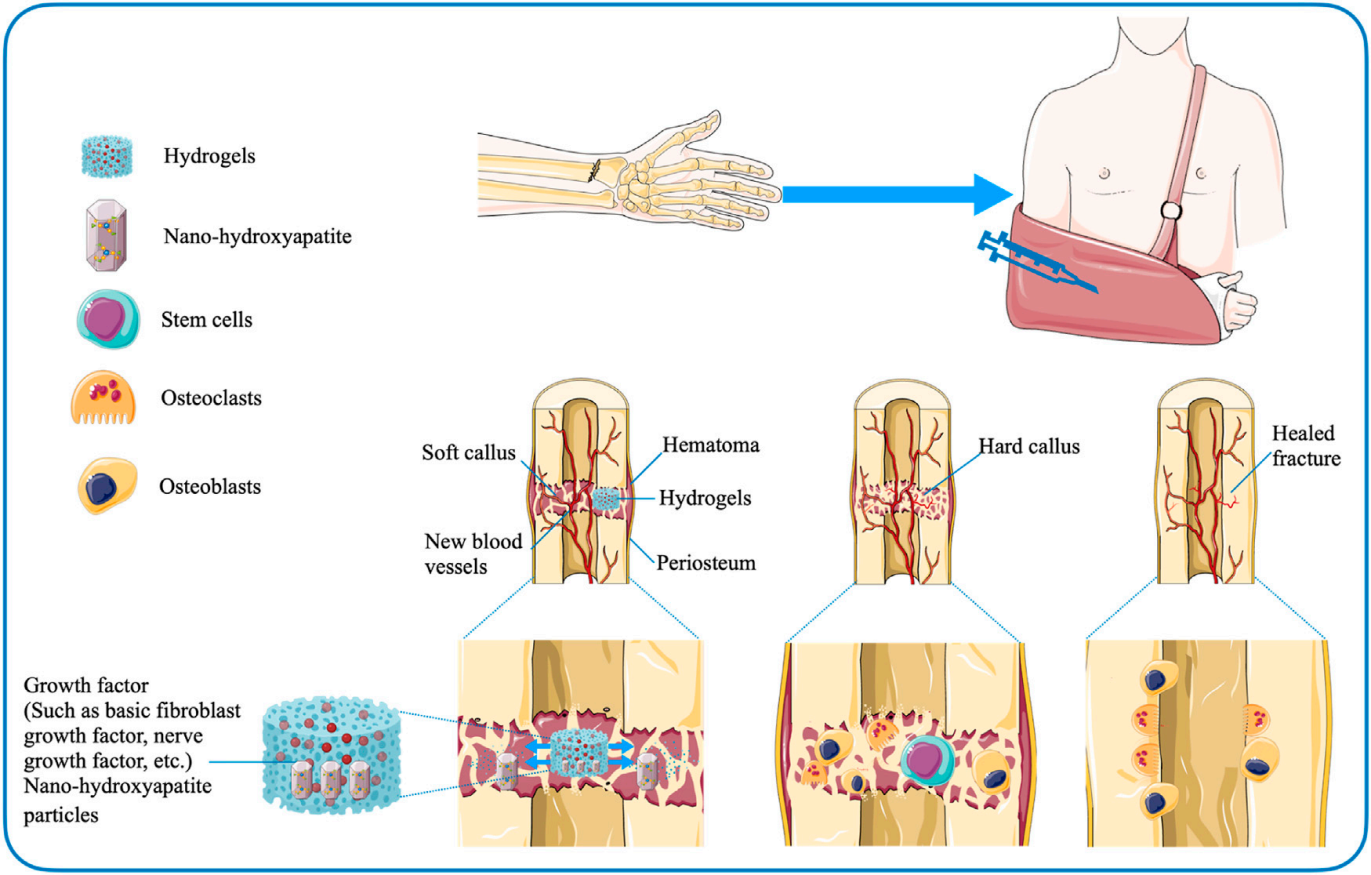
Hydrogel-mediated sustained release of growth factors accelerates fracture healing.

For patients with undisplaced fractures, who do not require bone grafting and are treated conservatively, injectable hydrogel-mediated growth factor delivery vehicles may be considered to accelerate fracture healing (Gibbs et al., 2016). Wang et al. (2008) investigated the ability of collagen-based gels containing nerve growth factor (NGF) and nano-hydroxyapatite particles to strengthen bone formation and explored the potential clinical efficacy of hydrogels in conservative treatment of fractures. The research team injected the gel into the callus of the rabbit mandible and found that the hydrogel facilitated growth factor-mediated osteogenesis (Wang et al., 2008; Gibbs et al., 2016). Sasaki et al. (2013) also proposed that a gelatin-based hydrogel containing basic fibroblast growth factor (bFGF) could boost the healing of fracture of the proximal sesamoid bone. The hydrogels are not only safely applied to the injured region, but also the level of degradation can be regulated by adjusting the degree of cross-linking (Sasaki et al., 2013) (Figure 4). In addition, the research data suggested that the mixed growth factor delivery system based on alginate and electrospun nanofibrous mesh-based hydrogels may promote bone regeneration during fracture nonunion, providing preliminary basic support for follow-up studies (Kolambkar et al., 2011). Regarding the development of nanotechnology, there are also studies to explore the effect of gelatin on the stability of silver nanoparticles (AgNPs) and the application of related polymers in fracture treatment. The experimental group prepared AgNPs-loaded Gel hydrogels under sunlight using gelatin as a stabilizer. The researchers found that the synthetic hydrogels were not harmful to osteoblasts. And they further effectively improved the survival rate and diffuse of osteoblasts, showing the potential ability to regulate fracture healing (Han et al., 2021).

It has to be mentioned that fracture repair is often accompanied by the risk of infection (Metsemakers et al., 2015). Prophylactic antibiotic therapy can effectively reduce the incidence of infection. However, edema, destruction of vasculature and tissue often limit the penetration of antibiotics, reducing the therapeutic efficacy. Fortunately, this conundrum can be overcome through the application of hydrogels. The carriers transport antibiotics to specific sites to exert their effects. Boo et al.(Ter Boo et al., 2018) demonstrated the great feasibility of the gentamicin-loaded hydrogel in preventing infection in a rabbit humeral osteotomy model. Moreover, Lu et al. (2018) proposed that copper-containing hydrogels also exhibited obvious antibacterial activity.

### 3.3 Cartilage damage

Damage to cartilage and osteochondral tissue is a common global public health problem. Its occurrence is closely related to diseases such as joint trauma, osteochondritis dissecans, and osteoarthritis. The prevalence rate of cartilage and osteochondral injuries in the general population is 60% ( Liu et al., 2017 ). Cartilage is avascular and lacks sufficient progenitor cells and nutrients to heal itself when damaged (Huey et al., 2012). If left untreated, cartilage damage will progress and become irreversible, leading to osteoarthritis that can eventually lead to disability (Chen et al., 2009). Current treatment strategies mainly include repair of microfractures, and autologous chondrocyte implantation, osteochondral autografts and allografts (Benazzo et al., 2008; Selmi et al., 2008; Hamblin et al., 2010; Haene et al., 2012; MacDonald et al., 2016; Polat et al., 2016; Gou et al., 2020). Despite their widespread usage, these approaches have significant drawbacks and limitations (Dai et al., 2020). At present, therapeutic techniques targeting cartilage lesions are difficult to cure cartilage damage, thereby accelerating the development of alternative tissue engineering strategies. Combined with BTE, it is a feasible idea to create artificial structures that mimic the structural characteristics, mechanical properties and biological functions of cartilage tissue. Cartilage tissue has high intensity, elasticity, and shock absorption (Pascual-Garrido et al., 2018). Consequently, it is promising to prepare high-intensitive, whippy hydrogels to mimic the mechanical characters of natural articular cartilage (Dai et al., 2020).

Regarding the physical cross-linking of hydrogels, although their effects on chondrogenic differentiation have rarely been investigated, some studies have considered the influence of the ionic effect of physical cross-linking of hydrogels on the biological behavior of cartilage. Xu’s group (Xu et al., 2018) developed a biomaterial that binds copper to promote cartilage formation. *In vitro* studies showed that Cu promoted morphological changes of MSCs, the production of glycosaminoglycan (GAG) and the expression of chondrogenic genes. To prepare hydrogels that can be applied to soft tissue, in the presence of positively charged quaternary poly (ethylene imine) (Q-PEI) and micelles formed by Pluronic F127 diacrylate, Mahapatra et al.(Das Mahapatra et al., 2020) prepared hydrogels with excellent tenacity by means of dual networks cross-linking. At the same time, this system also contains Ca^2+^ and Cu^2+^ ions, which form coordination bonds and effectively elevate the tensile strength and mechanical intensity of the hydrogels. Hydrogels with dual-ion cross-linked networks and hyper-extensibility have been successfully designed (Xue et al., 2021). In the field of chemically cross-linked hydrogels, Zhang’s research group (Zhang et al., 2020) designed a bi-component hydrogel based on HRP-induced cross-linking reaction, which is composed of collagen type I-tyramine (Col-TA) and hyaluronic acid-tyramine (HA-TA). The hydrogel possessed remarkable physicochemical properties and the conjugated TGF-β1 released from the hydrogel greatly promoted the BMSCs’ capacity of chondrogenic differentiation *in vitro*. Besides, *in vivo* experiments, histological and immunohistochemical analyses revealed that this enzyme-catalyzed hydrogel could exhibit the superior effects of cartilage repair.

Overall, hydrogels formed without covalency possess tunable mechanical performance and self-healing power, holding great promise in evaluating biomaterials for cartilage tissue regeneration. Combined with the rapid development of modern information technology, it is suggested that the introduction of 3D bioprinting in the field of hydrogel preparation may help meet the needs of patients. The biological structure can be quickly designed through automated and computerized technology to mimic natural cartilage tissue. The development of elastic and high-strength hydrogels for 3D printing in repairing cartilage defects and the osteochondral interface is crucial. It is extremely important to develop highly powerful and flexible hydrogels for 3D printing in repairing cartilage damage. This type of hydrogels may provide novel insights into treating cartilage diseases (Dai et al., 2020).

#### 3.3.1 Osteoarthritis

Joint cartilage is fragile and prone to damage. As mentioned above, cartilage tissue lacks blood vessels and has low metabolic activity, so the associated damage is usually irreversible (Zhang et al., 2016). Long-term damage to articular cartilage ultimately gives rise to the development of OA. OA is a prevailing degenerative joint disease. Its occurrence is highly correlated with the damage to cartilage structure and the up-regulation of permeability, mainly showing the characteristics of cartilage lesions (Hochberg, 2012; Zhang et al., 2016; Quicke et al., 2022). To date, the main treatment modalities for OA include lifestyle interventions, drug therapy (nonsteroidal anti-inflammatory drug, NSAID), and intra-articular therapy (Quicke et al., 2022). Current treatments are limited and only relieve symptoms (Zhang et al., 2008). There is an urgent need to find suitable treatments to impede the progression of OA (Zhao et al., 2022). Although the treatment of OA is hindered by the limitation of the anatomical structure of the joint cavity, the applicability of hydrogels provides new ways of thinking about it (Zhao et al., 2022). Hydrogels are elastic and adhesive, with a range of superior mechanical properties, making them particularly suitable for application in small and relatively isolated joint cavities (Kim et al., 2011). Hydrogels have promising applications in BTE as platforms for loading stem cells and medicines. With regard to hydrogels for OA treatment, there has been substantial research evidence of their effectiveness, accelerating tissue regeneration and the delivery of drugs.

The synovial joints carry a significant load on the human body and have extremely low friction under physiological stress when healthy. Normal frictional stress is typically required to maintain cartilage homeostasis (URBAN, 1994). When cartilage damage occurs (caused by sports, accidental trauma, or old age wear), the boundary layer on the outer surface of the cartilage is destroyed, resulting in dysfunction of cartilage lubrication and increased friction and wear. The limited self-healing ability of articular cartilage cannot cope with high friction, leading to the occurrence of OA, which is mainly characterized by progressive degeneration of articular cartilage (Sellam and Berenbaum, 2010; Goldring and Goldring, 2016; Morgese et al., 2018). In the treatment of OA, reducing friction between articular cartilage surfaces remains an important issue. Hydrogels with idealized mechanical properties and high water content, such as double-network hydrogels, contain highly hydrated lubricating carriers that can provide reservoirs to complement the boundary layer on the gel surface (Lin et al., 2020; Shoaib and Espinosa-Marzal, 2020; Liu Y. et al., 2021; Wang J. et al., 2021; Hilšer et al., 2021; Xie et al., 2021; Chen et al., 2022). These layers work through a hydration lubrication mechanism, resulting in extremely low friction, which is replenished when worn, providing long-term lubrication (Lin and Klein, 2022). It is reported that cartilage-lubricating brush-like polymers (hyaluronic acidgraft-poly-2-acrylamide-2-methylpropanesulfonic acid sodium salt (HA/PA) and hyaluronic acid-graft- poly-2-methacryloyloxyethyl phosphoryl choline (HA/PM)) could effectively combine on the cartilage surface to form a stable boundary layer *in vitro* and *in vivo*, which can lubricate and regenerate cartilage (Xie et al., 2021). On this basis, Chen et al. (2022) blended HA/PA and HA/PM (HPX) with polyvinyl alcohol (PVA) to construct biomimetic cartilage-lubricating hydrogels (HPX/PVA). The hydrogels exhibit low friction and wear, effectively addressing the main drawback of PVA hydrogels used as cartilage implants. It can be seen that the addition of HA/PA and HA/PM can improve the tribological properties and biomimetic properties of PVA hydrogels, which provides the possibility of introducing the boundary lubrication mechanism in the hydrogels (Rong et al., 2020; Lin and Klein, 2021; Branco et al., 2022).

**FIGURE 5:**
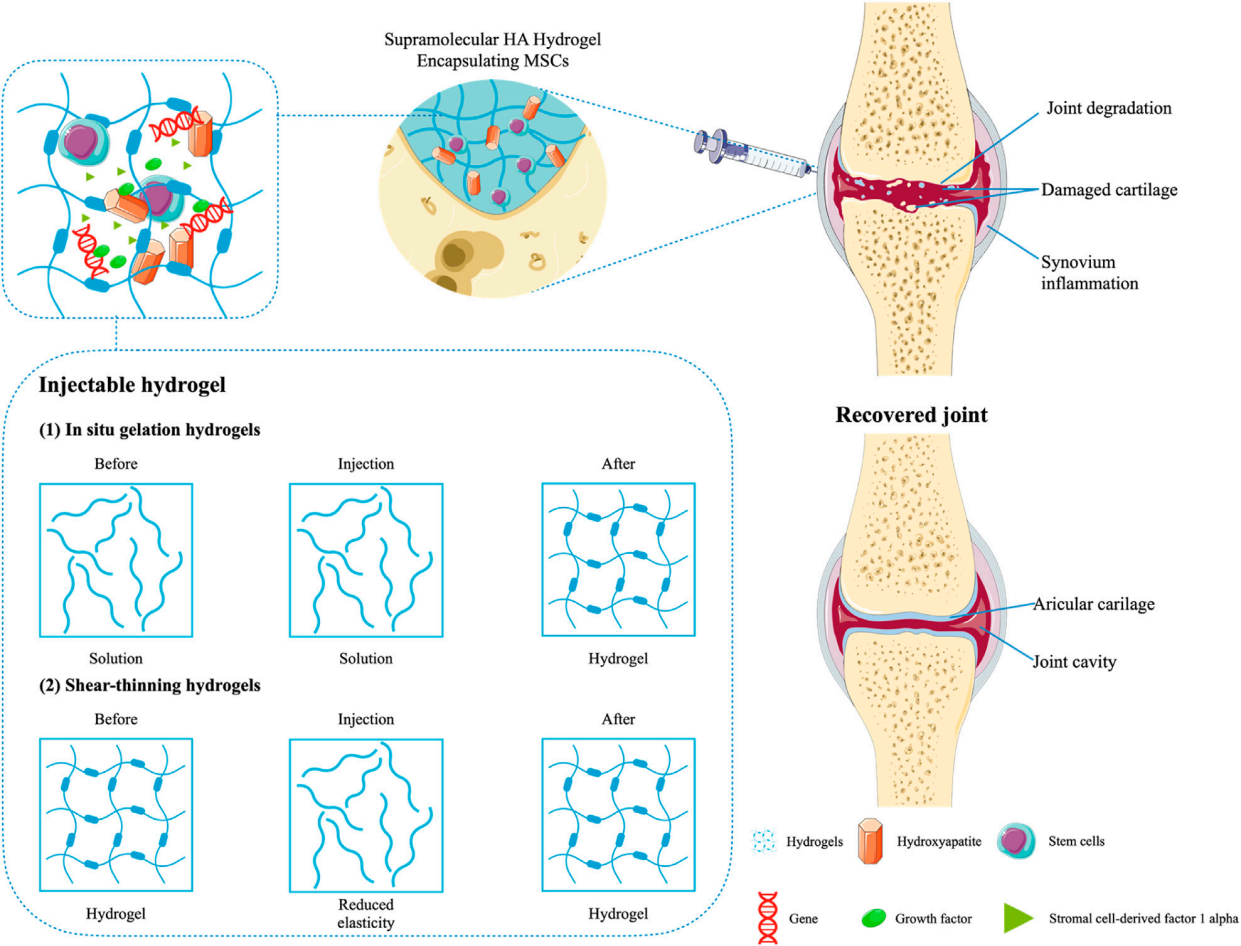
Injection therapy of hydrogels in OA.

Additionally, it should be noted that HA-based hydrogels have therapeutic efforts on OA, with data showing respectable pain relief by intra-articular injection of HA and chondroitin sulfate (Zhang et al., 2008). The hydrogels replenish joint fluid and reduce friction between articular cartilage surfaces. In order to improve the efficacy and retention rate of HA, it can be injected into the joint cavity together with the coupling with a thermosensitive polymer. The biocompatibility is maintained by reducing enzyme sensitivity (Maudens et al., 2018). One clinical study suggested that oral NSAIDs could be combined with intra-articular HA and corticosteroids in OA patients with persistent symptoms. Especially in the case of no reactions caused by other drugs, it was more applicable (Alexander et al., 2021). As mentioned previously, HA-based hydrogels are effective in relieving OA-related pain. This is because hydrogels with polyporous structures can expedite cell multiplication and tissue formation by slowly releasing drugs into the synovial cavity, ultimately suppressing inflammation and repairing cartilage damage (Jeuken et al., 2016). Not only that, the hydrogel scaffolds can also host cells and promote cell growth by transmitting signals and nutrients (Jeuken et al., 2016; Bao et al., 2020). For example, Thiolated gelatin/poly (ethylene glycol) diacrylate (PEGDA) interpenetrating network (IPN) hydrogels can simultaneously deliver progenitor cell populations and insulin-like growth factor-1 (IGF-1). By the way, the attachment of IGF-1 to the hydrogels can further support the long-lasting role of stem cells in their proliferation and the regeneration of tissue (Cho et al., 2020). Furthermore, in the treatment of OA, the formation of hyaline-like persistent cartilage is often promoted by implanting MSCs during surgery (Kristjánsson and Honsawek, 2014). Stromal cell-derived factor 1 alpha (SDF-1α) is a crucial factor in MSCs-related biological processes that involve activation, mobilization, homing, and migration of MSCs. Using a chitosan-based hydrogel inset with SDF1α to affect the migration of MSCs significantly promoted homing of the stem cells and repair of cartilage in the OA model (Liu H. et al., 2021). In addition, DNA supramolecular hydrogels are promising cell delivery systems for MSCs therapy, with significant protective effects on MSCs both *in vitro* and *in vivo*, which can be used to treat severe OA models. Studies have shown that DNA supramolecular hydrogels could promote the formation of high-quality cartilage under high-friction conditions of osteoarthritis (Yan X. et al., 2021) (Figure 5).

#### 3.3.2 Rheumatoid arthritis

**FIGURE 6:**
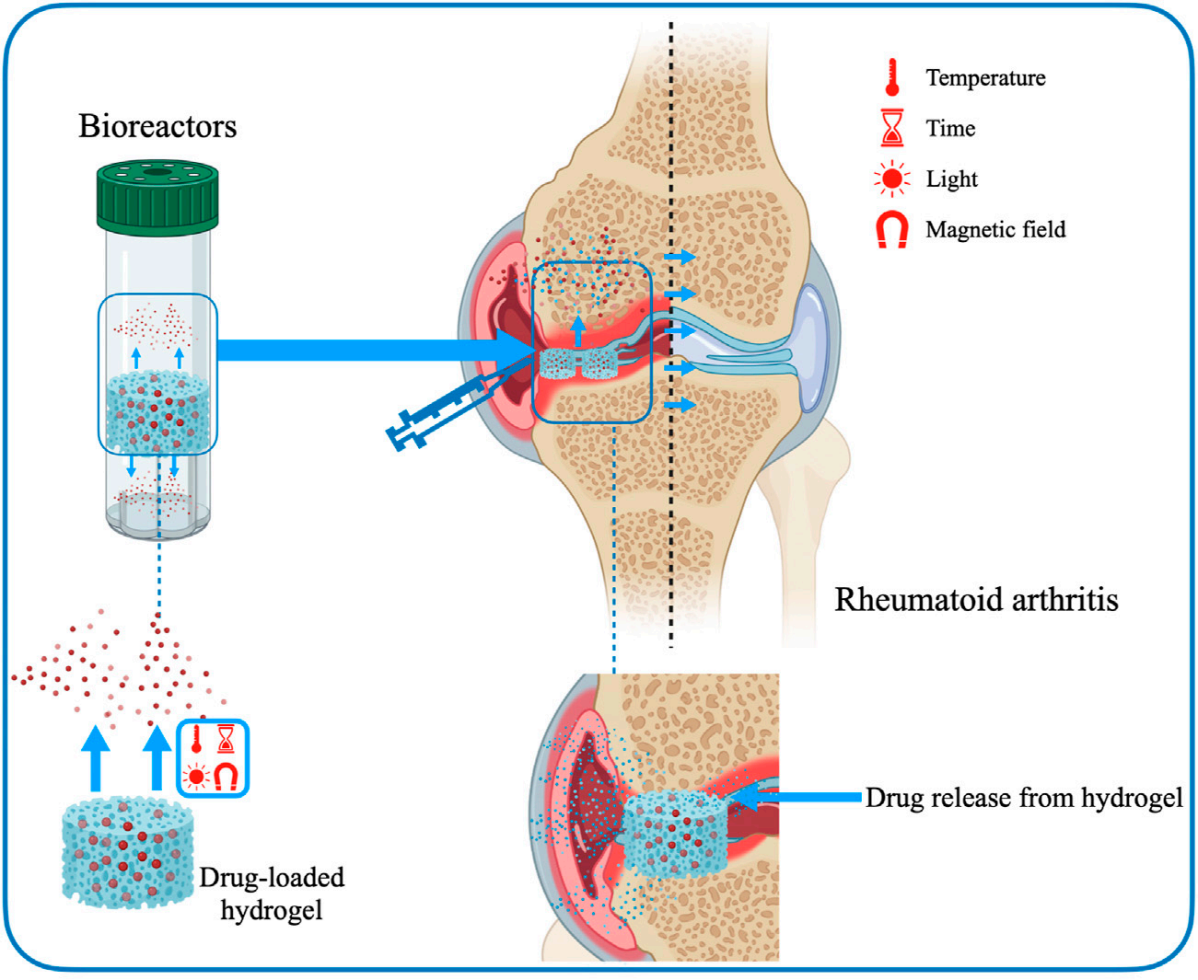
Drug delivery hydrogels in suppressing of RA.

RA is an autoimmune and chronic inflammatory disease that primarily affects articulationes synoviales and frequently involves injures to both arthroidal cartilage and bone (Hilkens and Isaacs, 2013; Oliveira et al., 2021). The definite pathological mechanism associated with RA remains unknown, but it is generally believed to be related to the breakdown of the state of immune tolerance (Weyand and Goronzy, 2020). Currently, several conservative treatments for RA are mainly used for pain relief and control inflammation. However, traditional modes of administration are not fully effective and have serious adverse side effects. Most immunomodulators suffer from deficiencies such as increased size, low stability, poor permeability to lesion sites and limited ability to cross cell membranes. Hydrogels can be used as carriers for drug delivery to effectively improve the therapeutic effect of biopharmaceuticals (Xiao et al., 2021). Hydrogels as drug delivery systems are a very attractive platform to ensure that these barriers are reduced and the therapeutic effects of drugs are maximized. Furthermore, hydrogels can mimic physiological microenvironments and possess the mechanical behaviors required for use as *in vitro* models of cartilage (Oliveira et al., 2021) (Figure 6). The specific advantages of hydrogels mainly include expanding blood circulation, promoting penetration of diseased tissue, improving accumulation, increasing uptake, enhancing drug-carrying capacity and being easy to modify physicochemical properties (Ahmed and Bae, 2016; Lu et al., 2016; Donahue et al., 2019; Su et al., 2019; Zhu Y. et al., 2020; He et al., 2020).

Tacrolimus-loaded soluplus hydrogel responds to sensitive temperature and is a hopeful vehicle of drug transport for RA therapy (Xue et al., 2021). Researchers used soluplus and tacrolimus to create a micelle-linked hydrogel for the treatment of RA (Wu et al., 2017). Combining the two components greatly modulated the drug release rate and improved the mechanical properties. Specifically, soluplus formed micelles by self-assembly and loaded tacrolimus. The hydrogel was formed under hydrophobic interactions, creating an internal environment with stable mechanical properties to store topical drugs. Furthermore, experiments in the rat models confirmed that the tacrolimus-loaded soluplus hydrogel has a better treatment effect on RA compared to the known poloxamer 407 delivery system.

### 3.4 Osteosarcoma

OS is the most common primary bone tumour among adolescents and children (Siegel et al., 2021). The traditional therapeutic method is surgical resection combined with chemotherapy regimens, but there are some limitations, leading to systemic side effects, postoperative recurrence, infection, and massive bone loss, while chemotherapy drugs have poor selectivity and drug resistance (Chen et al., 2021). Therefore, it is necessary to find new therapeutic strategies to improve the therapeutic effect and avoid any side effects. Nanoparticle-based drug delivery systems have been exploited to form a promising new-style alternative therapeutic strategy, which can both deliver drugs accurately to tumor sites and repair bone defects after tumor excision (Angulo et al., 2017; Chi et al., 2017; Corre et al., 2020; Yan J. et al., 2021; Gill and Gorlick, 2021; Zhu et al., 2021; Wu et al., 2022). Yin’s research group (Yin et al., 2020) developed a new-type nanoimplant SP@MX-TOB/GelMA with multiple functions. Under 808 nm near-infrared (NIR) irradiation, this effect under thermal ablation can efficiently remove OS cells and promote bone regeneration *via* hyperthermia. On the side, SP@MX-TOB/GelMA can carry high-efficiency antibacterial agents to prevent infection. In conclusion, this multipurpose implant under photothermal control can greatly eliminate OS cells, fight infection and enhance osteogenic ability (Wu et al., 2022). Although hydrogel nanoparticles have many benefits, their inherent properties also inevitably limit their clinical applications. For example, the specific hydrophilic nature of hydrogels may complicate the formulation of high drug loading and sustained drug release. For another example, studies have shown that nanoparticles with a diameter of more than 100 nm cannot form stabilized hydrogels; hence the diameter and amount of nanoparticles should be strictly controlled (Beckett et al., 2020; Wu et al., 2022). In the future, more work will be needed on the preparation.

## 4 Conclusion

At present, there are many opportunities to apply small molecule substances and biomaterials in clinical applications. It has been argued that the final objective is to ameliorate the biological response and rebuild nascent systems. On the long road to finding ideal biomedical materials used in clinics, efforts should be made toward composite hydrogels. For example, numerous biologically active substances possess osteoinductive properties that can be studied and analyzed in combination with hydrogels, mainly including steroids, collagen, casein phosphopeptides, prostaglandin agonists and amelogenin (Yue et al., 2020).

Hydrogels have been intensively studied because of their stability, but their preparation and application remain challenging by reason of the complicacy of the inherent properties of this class of materials. Numerous research groups have focused on developing new polymerization methods to synthesize polymers with different structures while avoiding using large amounts of harmful solvents. In this process, the molecular structures have been adjusted to design high-performance hydrogels required in specific circumstances, hoping that patients will benefit from them in the near future. Although the research results of hydrogels in BTE are gratifying, providing similar results to natural tissues, further research and development are still needed to seek the optimal research objects in each performance segment (aspects of physics and biology) and then enter into clinical translation (Nallusamy and Das, 2021).

It must be pointed out that there is still a long way to go to penetrate the market owing to insufficient clinical research evidence to prove the efficacy of hydrogels. The large-scale production of such new biomaterials faces technical and economic difficulties. It is predicted to be challenging to introduce them into the clinic and the market (Haugen et al., 2019; Liu et al., 2022). It needs to be emphasized again that improving the safety and adaptability of hydrogels is a pivotal issue that should be addressed in our future research. Conducting *in vivo* study designs is a critical and highly challenging part of testing biomaterials for safety and efficacy. The effect of hydrogels in BTE can be studied in relatively simple small animal models. Depending on different research purposes, the settings of the animal model can be adjusted and modified. However, final pre-clinical testing in larger animals should be performed to understand the suitability of the hydrogels and determine if it is conditioned for the optimal transition from the bench to the bedside.

Most of the research is only in the experimental phase and has not yet begun to be applied to the stage of clinical therapeutics. Here, the following recommendations are summarized. 1) Major factors such as hydrophilicity have been adjusted to design hydrogels with superior performance, but it is still essential to establish a complete set of methods to assess the biocompatibility of hydrogels with humans (Yue et al., 2020; Khiabani et al., 2021). 2) Many different hydrogels have been studied in animal models. However, Further standardization of animal models and procedures is needed to improve the comparability of studies to elucidate the therapeutic effects of hydrogels on bone-related diseases and assess safety and applicability (Kim and Kim, 2013; Nikravesh et al., 2020; Yue et al., 2020). 3) In bone-related diseases, many studies on hydrogels based on human models should be carried out (Xue et al., 2021).

As a new functional polymer material, the hydrogel has great application value in BTE. Understanding the properties, preparation and cross-linking methods of hydrogels can help us further grasp the progress of their applications in bone-related diseases. The idea of combining hydrogels with other biomaterials has gradually become a general strategy for treating bone-related diseases (Chen et al., 2016; Ghosh et al., 2018; Hu X.-B. et al., 2019, 4; Liu et al., 2022; Sun et al., 2022). Overall, though hydrogel-related biological materials are still under development and have many challenges, they undeniably have great potential in future clinical treatments (Yue et al., 2020). The application of hydrogels builds a bridge for therapeutics of bone-related diseases.
